# Quartz porosity in amorphous SiO_2_ of granitic shear bands

**DOI:** 10.1038/s41598-026-37576-x

**Published:** 2026-02-02

**Authors:** Jacques Précigout, Cécile Prigent, Gina McGill, Laurent Arbaret, Laura Airaghi, Mathieu Soret

**Affiliations:** 1 Institut des Sciences de la Terre d’Orléans (ISTO), Univ. Orléans, CNRS, BRGM, UMR7327, Orléans, France; 2 Institut de Physique du Globe de Paris (IPGP), Univ. Paris Cité, CNRS, UMR7154, Paris, France; 3 Laboratoire de Géologie, École Normale Supérieure (ENS) de Paris, CNRS, UMR8538, Paris, France

**Keywords:** Central cycladic detachment, Electron backscatter diffraction, Focused ion beam cross-sectioning, Geometrically necessary dislocations, Etch pits, Mechanical amorphization, Materials science, Solid Earth sciences

## Abstract

Important porosity is commonly observed in quartz-rich rocks that viscously deformed at depths of the metamorphic continental crust. Although the presence of such pores – often occurring with angular, pyramidal shapes – has major implications for fluid circulation, mass transfer and rock strength, whether or not they are directly produced by deformation remains unclear. Here we provide detailed observations of plastically deformed, pure quartz aggregates decorated by (sub)micrometric pores in granitic shear bands (western granite, Naxos, Greece). Using electron microscopy, we demonstrate that pores (1) decorate both grain boundaries and intra-grain substructures, (2) do not necessarily align with substructures when decorating grain boundaries, and (3) interconnect through layers of amorphous SiO_2_. Transmission electron microscopy further reveals dislocation densities one (or two) order(s) of magnitude below the predictions based on the lattice curvature gradients, suggesting the occurrence of residual stress along pore-decorated, partly amorphized substructures. Challenging long-lasting hypotheses, these features are here proposed to result from stress concentration at grain and subgrain boundaries, followed by fluid exsolution into mechanically amorphized quartz. Although involving syn-kinematic processes that remain to be fully understood, our findings corroborate recent studies that emphasise stress-induced amorphization as a general process of lithospheric rock deformation.

**Supplementary Information:**

The online version contains supplementary material available at 10.1038/s41598-026-37576-x.

## Introduction

Since the 19th century^1^, micrometric porosity has been regularly documented in quartz-rich, metamorphic rocks that experienced subsolidus viscous deformation. This porosity best occurs in quartz mylonite and quartzofeldspathic shear zones^2–6^, where pores decorate grain boundaries with various shapes, including pyramid-like ones commonly referred to as “etch pits”^7^. Based on dissolution experiments, the nucleation of such pores is generally attributed to preferential dissolution of dislocation traces during fluid infiltration through grain boundary interfaces^4,8,9^. However, field-based observations of quartz mylonites^3,6^ and statistical documentations of experimentally-produced porosity in Carrara marble^10^ suggest that micropores can also emerge from dynamic recrystallization by subgrain rotation, i.e., from viscous deformation. Recent evidence of micropores that locally decorate low-angle intra-grain boundaries^6,11^, which are not intuitively regarded as high-permeability conduits for fluids, further indicate that at least some pores are not the result of dissolved quartz. These features suggest instead that porosity results from processes other than preferential dissolution, possibly syn-kinematic and related to the dynamics of dislocations^11^.

Whether or not micro (to nano) pores directly arise from viscous deformation would yet bear important implications for deep geological processes. Pores indeed drive fluid percolation, local fluid pressure and fluid-rock interactions, contributing to mass transfer through dissolution-precipitation and possibly concentrating ore deposits^12–15^. Deformation experiments on calcite faults also indicate that embrittlement with coseismic implications is expected from pore accumulation in rocks that deform in the viscous regime^16^. Constraining how such porosity originates in natural rocks is therefore crucial for a better understanding of deep fluid circulation and related processes, as well as the seismogenic behaviour of the middle/lower crust.

In this study, we provide detailed documentations of (sub)micrometric pores that decorate grain and intra-grain ‘substructure’ boundaries in quartz-rich shear bands from the Naxos western granite (Greece)^11^. Using electron backscatter diffraction (EBSD) maps to document the kernel average misorientations (KAM) and geometrically necessary dislocation (GND) densities of quartz grains, we highlight numerous pores that do not locate at the intersection points between grain boundaries and substructures, which necessarily include dislocation traces. We then performed focused ion beam (FIB) cross-sectioning to provide three-dimensional views of pores at a voxel resolution down to 10^− 6^ µm^3^, documenting pore shape and distribution that do not support their production by preferential dissolution. Conversely, observations of FIB foils using transmission electron microscopy (TEM) revealed amorphous SiO_2_ along decorated boundaries, as well as dislocation densities of one (or two) order of magnitude below the ones predicted by EBSD analyses, giving us the opportunity to reconsider the source of porosity in deep crustal shear zones.

## Results

### Quartz microstructures and pore distribution in Naxos

**Fig. 1:**
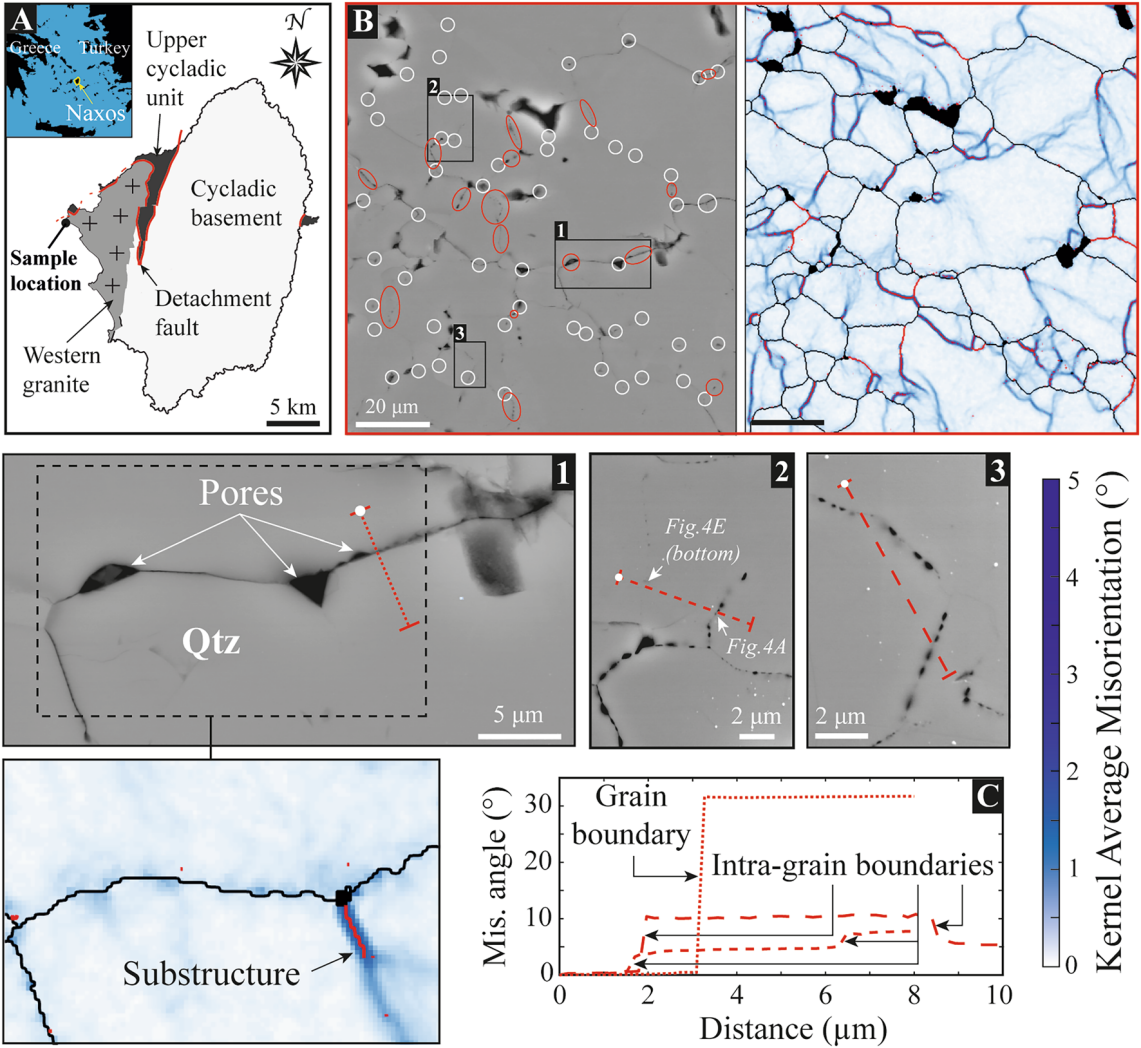
Pore distribution in pure quartz shear bands of the Naxos granite (Greece). **(A)** Location of the sampling area at the rim of the Naxos western granite nearby the central Cycladic detachment (red line). **(B)** Backscattered Electron (BSE) images and their companion EBSD maps (acquired at a step size of 0.25 μm; see the Method section) of a pure quartz shear band decorated by micropores. SEM images are located in supplementary Fig. 1. While white circles indicate the intersection points between grain boundaries and substructures, the red circles/ellipses highlight the sections of decorated grain boundaries that do not intersect with any substructure, as revealed by Kernel Average Misorientations (KAM). The parameters used to produce KAM maps are described in SI. Grain (black) and subgrain (red) boundaries respectively indicate point-to-point misorientations higher than 6° and between 2° and 6°. Inner boundaries are also revealed by KAM “lines” where subgrain boundaries are not present, i.e. where point-to-point misorientations remain lower than 2°. Close-up BSE and KAM images highlight pores along grain **(B**_**1**_**)**, inner **(B**_**2**_**)** and subgrain **(B**_**3**_**)** boundaries. **(C)** Transects of misorientation angle across the decorated (intra)grain boundaries shown in **B**. Misorientation angles are calculated for each EBSD point/pixel of the transect with respect to the first point (white dot on SEM images in **B**).

Located on the western side of the Naxos Island in the Aegean Sea (Greece), the western granite was emplaced and deformed below the top-to-the-north central-Cycladic detachment around 12 Ma ago (Fig. 1A)^17,18^. Pressure-temperature paths for the pluton indicate syn-tectonic decompression from 2.7 kbar to 1.5 kbar, and coeval cooling from suprasolidus conditions (∽650–680 °C)^19^ to the brittle-ductile transition (estimated at ∽350 °C), respectively preserved in the pluton core and nearby the detachment^20^. The deformation gave rise to multiple shear bands mostly composed of pure quartz aggregates and organized as S-C structures within a typical monzogranitic assemblage, including porphyroclasts of plagioclase and K-feldspar, as well as biotite flakes/ribbons and accessory minerals, such as amphibole^21^. Shear bands, which formed in the presence of H_2_O-rich fluids^19^, are increasingly well developed with distance from the core to the rim of the pluton and peak nearby the detachment where they are locally overprinted by brittle structures^21,22^. Accordingly, quartz is described by crystal plastic deformation dominated by recovery processes progressively changing from grain boundary migration to subgrain rotation with decreasing distance from the detachment^11^. Evidence of four-grain junctions and partial randomization of lattice preferred orientation also suggest a contribution of grain boundary sliding (GBS) to accommodate quartz plasticity, particularly in the “subgrain rotation” domain^11,21^.

Most of the porosity occurs in mylonitic quartz-rich shear bands, particularly where quartz recrystallization is dominated by subgrain rotation. Pores have a circle equivalent diameter ranging from a few nanometres to more than 1 micron with a mean size of ∽340 nm, and they have been identified along both grain and subgrain boundaries using EBSD^11^. Based on additional EBSD data and plotting KAM maps to reveal substructures, we confirm that pores are present along low-angle intra-grain boundaries (Fig. 1). This happens even by considering point-to-point misorientations between 2° and 6°, instead of the threshold angle of 10° classically used to define high-angle grain boundaries^23^. We did so in order to make sure that only intra-grain boundaries are considered to be compared with pore distribution (see the Method section). Furthermore, while many pores – often exhibiting angular shapes – occur at the intersection between grain boundaries and substructures, number of them decorate grain boundaries that do not intersect with any intra-grain substructure, at least in two dimensions (Fig. 1B and supp. Fig. 1).

**Fig. 2:**
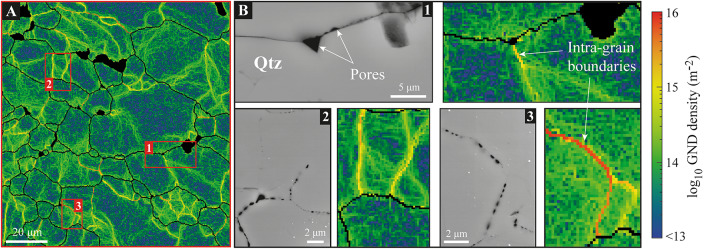
Geometrically Necessary Dislocation (GND) densities of the pore-decorated quartz-rich shear band. **(A)** GND densities calculated from the EBSD maps shown in Fig. 1B. GND densities are deduced from the lattice curvature gradients of quartz grains, using the KAM map parameters for denoising and a Poisson ratio of 0.1 (see the Method section). **(B)** Close-up GND maps and their companion BSE images of decorated grain **(B1)**, inner **(B2)** and subgrain **(B3)** boundaries.

The documentation of numerous intra-grain boundaries indicates significant plasticity of quartz grains that we then better characterized through estimates of GND densities (Fig. 2). Assuming that the lattice curvature gradients only result from crystal plasticity, we estimate densities lower than 10^13^ m^− 2^ in dislocation-poor areas (i.e., the noise floor of EBSD map produced with a step size of 0.25 μm^24)^ and densities ranging between 10^14^ and 10^16^ m^− 2^ along substructures, particularly along subgrain boundaries where densities are remarkably high (> 10^15^ m^− 2^). These values do not change significantly when improving the angular resolution of EBSD acquisition from 0.1° to 0.01° using high-angular resolution (HR-)EBSD^24^. On the contrary, computational post-treatment based on the cross-correlation of diffraction patterns, as applied for HR-EBSD, was even not possible where the curvature gradients were too high, including along some subgrain boundaries (supp. Fig. 2). This feature points to a prediction of very high dislocation densities along and nearby pore-decorated substructures.

### FIB cross-sectioning and TEM observations

**Fig. 3:**
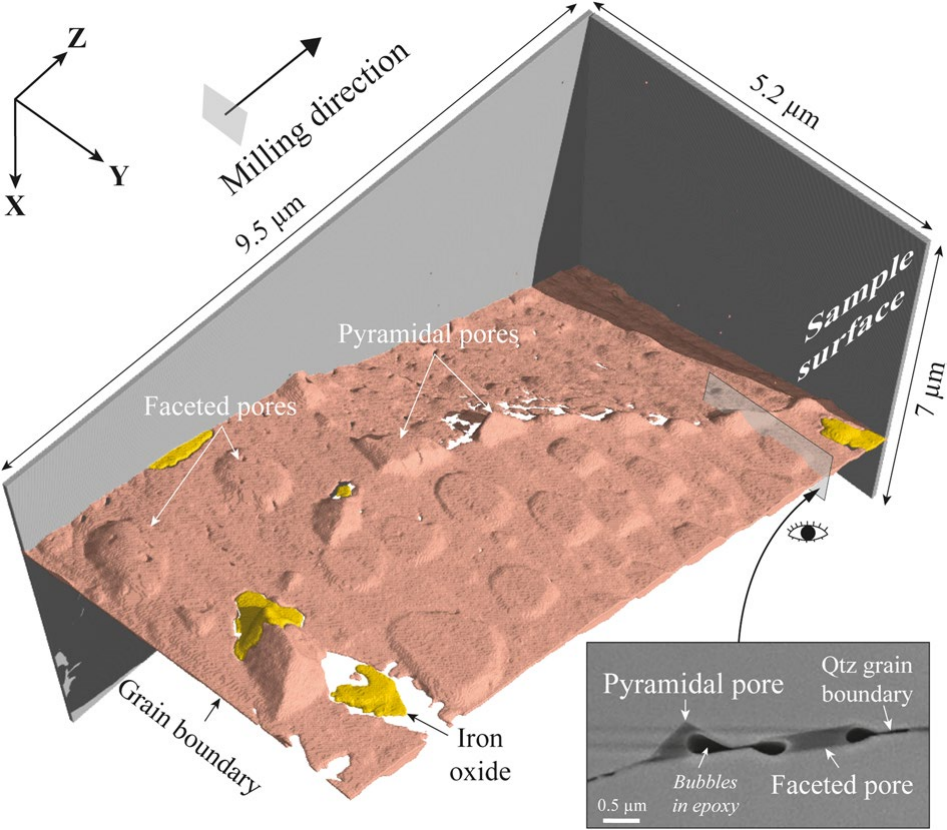
High-resolution, three-dimensional view of grain boundary porosity. Volume reconstruction of a decorated grain boundary using stacked SEM images. The latter have been acquired every ∽10 nm during Focused Ion Beam (FIB) cross-sectioning of an open grain boundary filled by epoxy, which contains bubbles and iron oxide (due to sample preparation). The FIB area is located in supplementary Fig. 6. SEM images have been then segmented and compiled using the VG-studio software, producing a three-dimensional view of pores with a voxel resolution of 10 × 10 × 10 nm^3^. The FIB volume highlights two different shapes and distributions of pores: (1) pyramidal pits that align with each other along interconnected traces, giving rise to a truncated pyramid at a triple point; and (2) pancake-like ‘faceted’ pores that do not align with each other and display opposite facets parallel to the boundary plane. The close-up section (bottom right) provides a SEM image (secondary electron/ion) of pores with either pyramidal shapes or parallel crystal facets at opposite sides of the boundary. Horizontal artefacts (which were actually vertical during the milling) are due to a curtaining effect. X = shear direction; Y = vorticity axis; Z = Pole of the shear plane.

To provide a detailed view of pore shapes, we performed focused ion beam (FIB) cross-sectioning along all types of quartz boundaries (supp. Fig. 3). Most of the FIB volumes have been reconstructed based on SEM images acquired every 100 nm to 200 nm with a pixel resolution of 10 × 10 nm^2^ (see the Method section and videos in SI). Along all sections, pores have been observed at grain and intra-grain boundaries, irrespective of pore size. They occur with different shapes, including lens-like, ellipsoid and geometric ‘pyramidal’ ones, as commonly described in quartz mylonite *sensu lato*^3,4,25^. Pores have angular boundaries that may locally combine with rounded ones and they include trapezoid pyramidal pits that strongly resemble pores interpreted as etch pits in previous studies^4,9^. In addition, through reconstruction and segmentation of SEM images acquired every 10 nm, producing a FIB volume with a voxel resolution of 10 × 10 × 10 nm^3^ (Fig. 3), we provide highly detailed views of the pore geometry in three dimensions (3D). While pits align along traces that connect at a triple point where a truncated triangular pyramid occurs, their tip always points to the same side with respect to the boundary. However, the 3D volume also reveals faceted pores that do not align with each other and adopt pancake-like shapes with parallel crystal facets at opposite sides from the boundary. They all have the same geometry, the section of which is close to hexagonal and quasi-symmetrical to the boundary plane (Fig. 3). They roughly display the same depth of penetration irrespective of pore size, which does not correlate with the distance to the pyramidal pits.

**Fig. 4:**
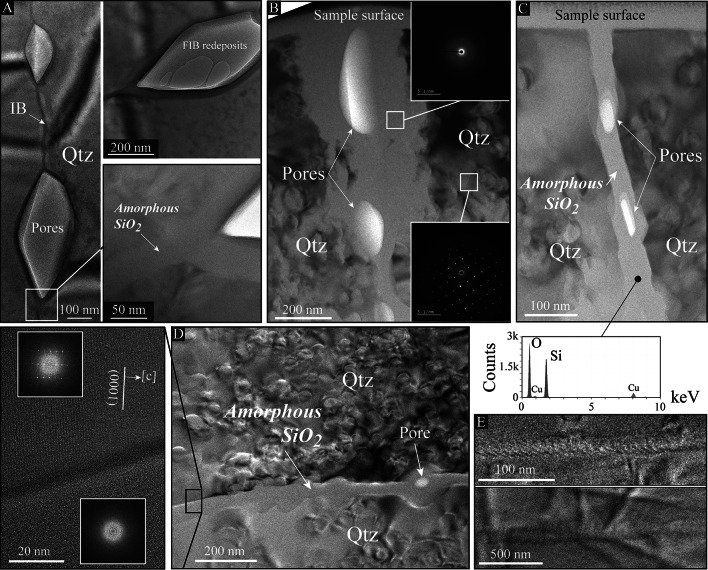
Evidence of amorphous SiO_2_ at (intra)grain boundaries. **(A-C)** Bright-field TEM images across intra-grain boundaries decorated by micropores. TEM observations have been performed on FIB foils located in Fig. 1 and supplementary Fig. 1. While pores adopt various shapes, including angular ones (**A**), they are embedded into amorphous SiO_2_, such as indicated by spotless diffraction patterns (subsets in **B**) and energy dispersive spectroscopy (spectra in **C**). FIB redeposits may also have occurred in pores during the milling, as observed in **A**. IB = Intra-grain Boundary; Qtz = Quartz. **(D)** Bright-field TEM images across a grain boundary. While quartz crystals are highly contrasted by loop-shape structures (probably because of local damage during the FIB milling; see the Method section and supplementary Fig. 4), amorphous SiO_2_, embedding a micropore, is here again observed and highlighted using a lattice fringe image (close-up) together with Fast Fourier Transform (FFT) diffraction patterns (subsets) across the boundary. **(E)** Bright-field TEM images of a grain (top) and an intra-grain (bottom) boundaries, both highlighted by Moiré interference patterns. This feature indicates that the boundaries do not contain any – or contain very little – amorphous SiO_2_.

**Fig. 5:**
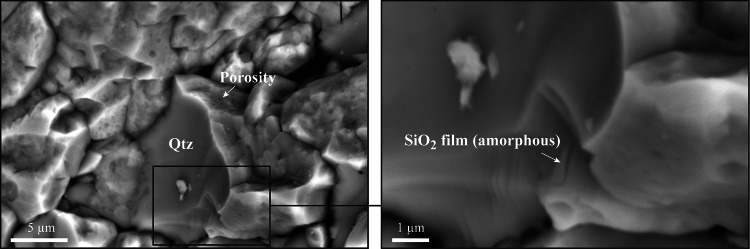
SEM images (backscattered electron) of a quartz-rich shear band on a broken surface. While quartz (Qtz) appears as dark grey, pores are embedded into a brighter film of SiO_2_, suggesting a different state (amorphous). Except for a 20 nm carbon coating, no sample preparation or any other analyses has been performed before SEM observation, here acquired approximately along the YZ section.

We then extracted FIB foils to observe the porosity using TEM across grain and intra-grain boundaries (FIB foil locations can be seen in Fig.1 and supp. Fig.2). Within grains, the crystallinity of quartz is attested by contrasted images and the presence of crystalline diffraction patterns (Fig.4A-D). Although dislocation traces have been observed (supp. Fig.4), TEM observations did not provide any evidence of high dislocation densities (< 10^14^ m^−2^) at the proximity of decorated grain or intra-grain boundaries. In contrast, (intra)grain boundaries do not diffract, but do contain pores – including angular ones – separated by hundreds of nanometers and fully or partly embedded into an amorphous layer of around 50 nm thick or more. While energy dispersive spectroscopy indicates that the amorphous material is only composed of SiO_2_ (Fig.4C), the amorphous state is here demonstrated by uniform, spotless diffraction patterns either revealed by using a magnetic lens (Fig.4B) or by producing Fast Fourier Transform patterns across a lattice fringe image of the layer (Fig.4D). Both, pores-bearing and pore-free (intra)grain boundaries have been identified as wetted by amorphous SiO_2_, but only pore-free ones may contain no amorphous layer, as attested by moiré interference patterns (Fig.4E and supp. Fig.4). The latter results from crystal lattices that overlap through the FIB foil thickness, indicating that no – or very little – amorphous SiO_2_ is present along the boundary. Amorphous SiO_2_ may be also identified using SEM to image broken surfaces of the shear band, i.e., before any sample preparation or FIB/TEM investigations. As already suggested by Mancktelow and Pennacchioni^25^ in quartz-feldspar mylonites, pores are indeed embedded into a film of SiO_2_ that arises as slightly brighter than quartz using secondary or backscattered electron detectors (Fig.5). TEM images furthermore revealed highly contrasted quartz crystals with numerous irregular loop-shape microstructures, a feature which probably results from the FIB milling because of local damages of the foil surface (see the Method section and supp. Fig.5 in SI).

## Discussion

The presence of pores adopting angular shapes at grain boundaries is a common feature of quartz mylonite, now described for more than one century^1–4^. Upon several campaigns of investigation, including field studies and dissolution experiments, such porosity has been regularly attributed to the circulation of fluid(s) through open grain boundaries and coeval preferential dissolution of quartz where dislocation traces intersect with the boundaries, giving rise to etch pits^4,8,9,26,27^. Although we do not exclude that the production of etch pits may occur in nature, we here provide new features that do not support this hypothesis for quartz mylonite, including for quartz-rich shear bands in granitic environment. At first, we show that pores do not necessarily coincide with the intersections between dislocation traces and grain boundaries. One may suggest that substructures might have subsequently migrated or recovered, so that pits do not intersect with them anymore, but we would expect pores to be systematically aligned with each other within the grain boundary interface. Although such an alignment is observed for pyramidal pits, this is not the case for a significant population of faceted pores.

The occurrence of angular pores decorating closed boundaries, as well as intra-grain substructures, does not support either the model of dislocation-driven preferential dissolution, particularly considering that massive fluid flow is required for etch pits to be produced. Indeed, dissolution experiments have shown that etch pits only occur when the concentration of solute (C_solute_) is far below the equilibration concentration (C_0_) of a H_2_O-rich fluid (C_solute_ = 0.75C_0_^7^. If such a fluid is present at closed grain boundaries or within grains in natural conditions, we expect it to be quasi-equilibrated with the surrounding quartz grains (i.e., C_solute_ ≃ C_0_), and hence, typically not in favourable conditions to produce etch pits. Furthermore, we here demonstrate that pores are connected to amorphous SiO_2_ along grain and intra-grain boundaries of pure quartz aggregates. Although rarely documented, the presence of amorphous layers in subsolidus metamorphic rocks has been already observed along both, grain and subgrain boundaries^9,28^, sometimes with evidence of porosity^29^. Based on chemical weathering models^30,31^, such a “metamorphic” amorphous phase has been attributed to either leaching or interfacial dissolution-precipitation processes^29,32^. However, little evidence of chemical weathering producing in-amorphous-layer (i.e., internal) porosity has been reported^33^; pores either occur within the dissolving mineral phase or are included into the secondary crystalline one. Moreover, to our knowledge, no amorphous material has been documented from dissolution experiments on pure quartz crystals, despite hundreds of experiments^34^.

Alternatively, quartz has been known for decades to amorphize under shock compression or very high confining pressure (several GPa), which has been demonstrated through diamond-anvil-cell and indentation experiments, as well as natural observations of meteoritic impacts^35–38^. Such conditions are obviously not relevant for the Naxos granite, but recently, Li et al*.*^39^ and Idrissi et al*.*^40^ have gathered data on deformation experiments that produced an amorphous material without any shock or high confining pressure. Amorphization was generated by applying a differential stress to subsolidus viscous materials, including industrial metals and alloys^39^, as well as representative crystals of natural rocks, such as quartz and olivine^40–42^. Irrespectively of the confining pressure and use of various types of triaxial apparatuses, these experiments were able to mechanically produce an amorphous phase, mostly in the form of a layer at grain boundaries, but also as a silica gel – or amorphous nanopowder – in case of room-pressure rotary shear experiments^43–45^. Although requiring further work to be fully understood, this process of mechanical amorphization has been so far attributed to either stress concentration at grain boundaries^38,42^ or crystal plasticity because of extremely high dislocation densities^46^ (10^16^−10^17^ m^− 2^).

In relation to this, GBS has long been considered and observed to be associated with the production of porosity during deformation experiments, the best examples of which are given for metals and ceramics^47–50^. These studies have depicted GBS to produce cracks at grain boundaries, sometimes in combination with dislocation tangles, giving rise to local dilatancy with the potential ability to pump the surrounding fluids^51^. As GBS highly contributes to the deformation of viscous shear zones where pores and fluid pumping have been evidenced, this process is regularly proposed to account for the production of porosity in deep environments^12,52,53^. Previous studies on quartz mylonites have also reported a significant contribution of GBS to deformation in zones where micropores occur^3^, including in Naxos^11,21^. Cracking, however, is not expected to produce any amorphous SiO_2_ around micropores, neither along grain boundaries nor *a fortiori* along intra-grain boundaries. Neither does it account for the presence of lens-like and ellipsoid pore shapes. Moreover, most of the porosity in Naxos is not observed inside grains, but along grain boundaries, and TEM observations do not show high dislocation densities at the vicinity of amorphous layers, which should be expected for healed cracks^54^. Interestingly, although not observed at the TEM, our EBSD data yet predict such high (to very high) dislocation densities along subgrain boundaries. As GND calculations estimate the minimum of dislocations required for a lattice curvature gradient to be plastically consistent (see the Method section), this feature indicates that crystal plasticity cannot account by itself for the lattice curvatures documented at the vicinity of decorated substructures. This suggests instead the occurrence of residual stress, probably elastic and potentially frozen by the presence of amorphous SiO_2_.

Regarding the occurrence of amorphous SiO_2_, the absence of high dislocation densities in our samples rules out quartz amorphization by intense crystal plasticity through dislocation accumulation^46^. Nevertheless, deformation experiments coupled with TEM observations have demonstrated that an amorphous material – including SiO_2_ – can form from stress concentration without producing any “extra” dislocation densities in the surrounding crystal^38,40,55^. Some of these experiments have even produced an internal porosity where stress-induced amorphization has been described, i.e., along grain boundaries^42^. In this case, pore nucleation has been attributed to cavitation induced by local strain incompatibilities^40,42,56^, but this hypothesis does not account for the presence of pores along intra-grain boundaries, as observed in Naxos. It is also noteworthy to mention that any amorphous material, including amorphous SiO_2_, has a strong potential to dissolve fluid, especially H_2_O^57^ (up to several wt%), the maximum concentration of which increases with increasing pressure/stress^58^. The source of H_2_O in the Naxos granite remains nevertheless poorly constrained, but the quasi-absence of local contamination at grain boundaries of quartz aggregates, where only Si is detected, suggests that any infiltration of an external fluid, if relevant, was very limited. Another possibility could be that H_2_O was drained from grain interiors to grain boundaries in response to crystal plasticity. Such a mechanism, recently highlighted experimentally^59^, could account for several percents of fluid(s) at grain boundaries, even by considering that quartz grains initially contained a few hundreds of ppm.

**Fig. 6:**
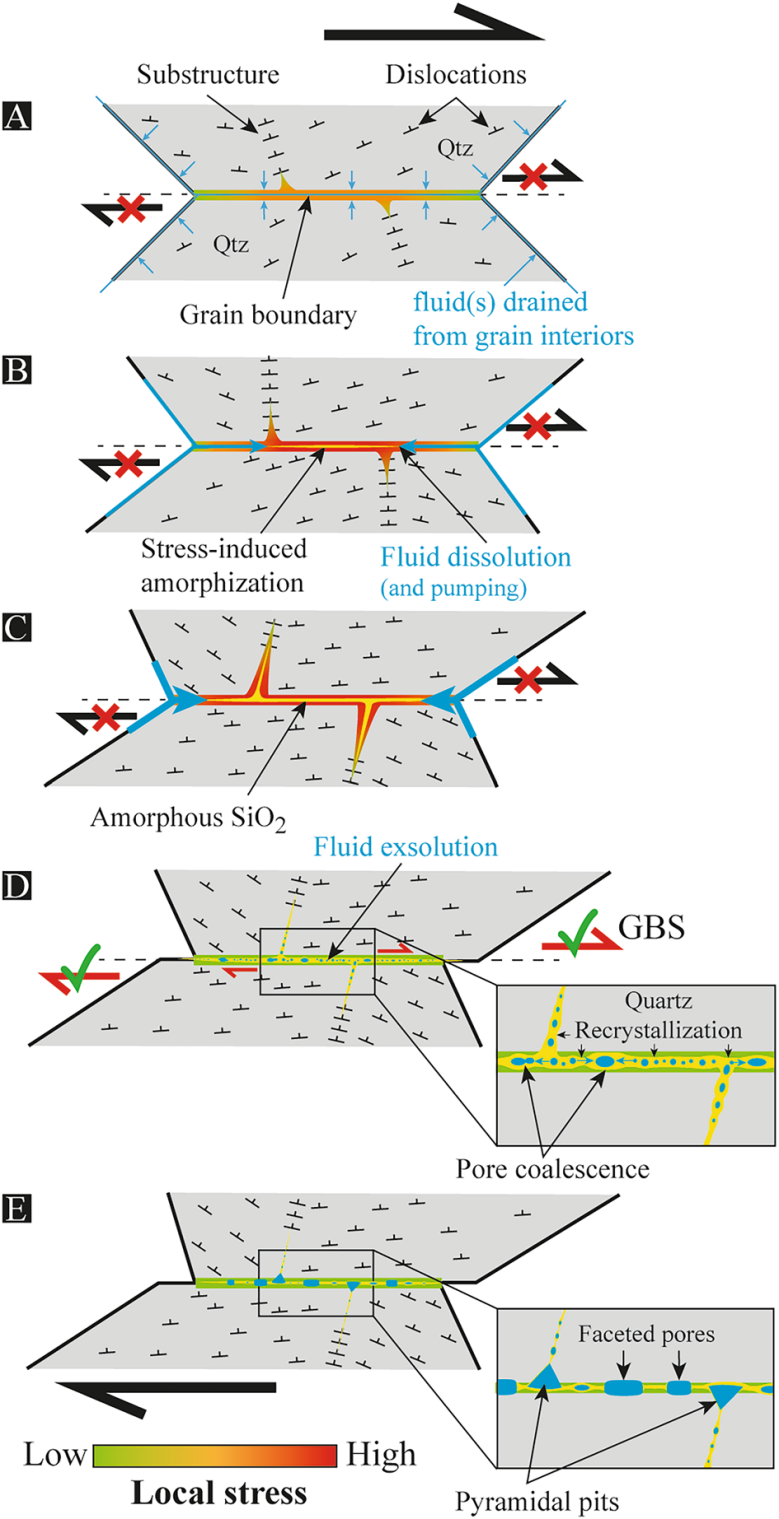
Model of porosity resulting from stress-induced amorphization**.** **(A-C)** Shear deformation and progressive drainage of fluid(s) from grain interiors to grain boundaries due to crystal plasticity. Because of stress concentration at grain boundaries, which might also expand locally into some intra-grain boundaries, quartz then amorphizes to produce amorphous SiO_2_. Fluid (probably H_2_O-rich) is also expected to be partially dissolved (and pumped) into amorphous SiO_2_ during amorphization. Qtz = quartz. **(D-E)** Grain boundary sliding (GBS) induced by full lubrication of the grain boundary, giving rise to a local stress drop and the coeval exsolution of the fluid to produce an internal porosity. Pores/bubbles are then proposed to grow because of (1) pore coalescence and/or (2) quartz recrystallization, until they get large enough to interact with the surrounding crystals, giving rise to crystallographically-controlled negative shapes. The latter (pyramidal vs. faceted) depends on their location with respect to the substructures.

On this basis, we tentatively propose an alternative model to account for the presence of (angular/faceted) porosity in amorphous SiO_2_ of quartz-rich shear bands in Naxos. We hypothesize that quartz first deformed plastically, promoting a fluid drainage from grain interiors towards grain boundaries. An amorphous material is then produced by stress concentration at grain boundaries, here attributed to the declining activity of quartz plasticity as the pluton of Naxos is cooling down during unroofing and deformation (Fig. 6A-C). As the shear stress increases, amorphous SiO_2_ progressively wets the grain boundary until full lubrication, promoting GBS and a coeval local stress drop (Fig. 6D). During amorphization, free volatiles are first dissolved into the amorphous layer due to stress concentration, and are then exsolved while stress is released. The term “exsolution” is here preferred to “cavitation”, because fluid bubbles are not envisaged to result from a phase transition during short decompression, but instead from a long-term instability of a fluid-saturated material. In this view, two possibilities may be anticipated to exsolve the fluid: it can result from (1) GBS due to a significant stress drop, as proposed for olivine^40,42^; and/or (2) recrystallization into quartz, such as demonstrated along planar deformation features in shocked quartz^60^. In both cases, the pore size is hypothesized to exceed the thickness of the amorphous layer, either by pore/bubble coalescence or due to progressive bubble growth as quartz is recrystallizing (Fig. 6D). In addition, depending on how intra-grain boundaries are oriented with respect to the local stress field, they may act as planes of weakness where stress has laterally propagated to amorphize them.

The shape of angular pores is very likely to have been controlled by crystallography^2,7^, and preferential dissolution driven by dislocation traces is one possible explanation^2,4,8^. This hypothesis does not however account for angular pores that we found along substructures, nor for the development of pancake-like faceted pores documented along grain boundaries. Alternatively, considering fluid inclusions that emerge from amorphous SiO_2_ by exsolution, fluid/mineral texture equilibrium may be envisaged as a first-order control of pore shape^25^. Fluid (or melt) inclusions with crystallographically-controlled shape are indeed commonly observed in various minerals, including quartz^61,62^. These negative crystal shapes are attributed to a minimization of surface energy under favourable conditions, implying relatively high temperatures typical of metamorphic or magmatic ones. In this case, pyramidal pore shapes would result from surface tensions driven by dislocation traces where the latter intersect with grain boundaries, whereas faceted ones would be controlled by the quartz lattice in-between dislocation traces (Fig. 6E). Considering our syn-kinematic model to produce a porosity, we might finally expect pores to preferentially decorate grain boundaries oriented quasi-parallel to the vorticity axis, i.e., the [0001]_c_ axis in Naxos^11,21^. Such a pore distribution has been recently highlighted in the Harkless quartzite by Nagurney et al*.*^27^, although attributed to the quartz alpha/beta transition in this study.

Our findings therefore provide detailed documentation of naturally-produced porosity embedded into layers of amorphous SiO_2_ at (intra-)grain boundaries. Based on evidence of residual stress, we propose that pores result from stress-induced amorphization followed by fluid exsolution from the resulting amorphous layer. Beyond the origin of porosity in deep crustal rocks, such a mechanical amorphization is here emphasised as a general process of rock deformation with strong implications for geological processes, as already reported for olivine^42^. Amorphous SiO_2_ has indeed a relatively low viscosity and may serve as a lubricant at grain boundaries for faults and shear zones far below the rock solidus^43,44^. Because of its high capacity to dissolve fluids, an amorphous phase is also anticipated to promote massive dissolution of volatiles and subsequent fluid pumping where the phase is produced, mostly in shear zones. Finally, successive cycles of local stress accumulation followed by fluid exsolution are finally predicted to trigger rock failure by amassing fluid-filled pores, particularly at the brittle-ductile transition zone^16^.

## Method

### Scanning electron microscopy

Scanning electron microscope (SEM) images and electron backscatter diffraction (EBSD) data have been acquired on a polished thin section using a FEG (field-emission-gun) SEM from JEOL (IT800SHL) coupled to an Oxford Instruments EBSD system with advanced Symmetry detector. The thin section has been produced from a rock slab cut perpendicular to the foliation (Z) and parallel to the lineation (X), and then glued with epoxy on a glass slide before thinning and polishing it to around 40 μm thick. Sample preparation and SEM/EBSD acquisitions have been performed at the *Institut des Sciences de la Terre d’Orléans* (ISTO) and MACLE-CVL platform of the *Centre National de la Recherche Scientifique* (CNRS) in Orléans (France), respectively. For SEM and EBSD analyses, the beam current/accelerating voltage were respectively set to 4 nA/15 kV and 6 nA/30 kV. EBSD acquisition has been performed using the Aztec 6.0 software from Oxford, applying a speed-1 resolution mode (image size of 622 × 512 pixels), a gain of 1 and a frame averaging of 3. For instance, using the indexing mode “optimized Band Detection”, the map in Fig. 1B has been acquired with a mean average deviation (MAD) of 0.75° and a hit rate of 90.8% before cleaning.

After acquisition, the EBSD map was first cleaned using the Oxford AZtecCrystal 2.2 software by (1) removing wild spikes of one pixel size and (2) replacing zero solutions by the mean orientation of correctly indexed neighbour pixels. Four steps of zero solutions removal have been applied, including 2 steps considering 6 neighbours and 2 other steps based on 5 neighbours. The second correction is particularly needed for grain boundaries to be filled, because the superposition of crystal lattices over the boundary produces diffraction patterns that do not satisfy the criteria imposed by AZtecCrystal for a solution to be valid. Criteria include, in order of importance, MAD, extended band matching, number of quadruplets and number of Kikuchi bands. After cleaning, the final hit rate of the map in Fig. 1B was 96.9%. The data were then exported and treated/plotted using the open-source MTEX toolbox (version 5.8.2; https://mtex-toolbox.github.io) for Matlab^63,64^.

### Geometrically necessary dislocations

Dislocation densities can be estimated from the geometrically necessary dislocations (GND), which are calculated from the lattice curvature gradients based on EBSD data^65^. This method offers the advantage of documenting dislocation densities with a statistical approach over representative areas, as recently confirmed for high-temperature quartz (> 550–600 °C) using HR-EBSD along chessboard subgrain boundaries^24^. However, GND analyses do not provide the total dislocation densities as they include statistically stored dislocations (SSD) that do not contribute to the lattice curvature over the length scale of observation^66^. Furthermore, as two-dimensional EBSD maps cannot measure orientation gradients in the direction normal to the specimen surface, dislocations required to generate these orientation gradients are omitted from the estimated GND densities. These effects mean that only the lower bound of dislocation densities in the deformed material is accessible. Although acquiring an EBSD map with a small step size (i.e., with a small Burger circuit) minimizes the contribution of SSD, GND densities thus necessarily underestimate the real number of dislocations in a given material.

In addition, rather than 10°, we chose to use 6° of misorientation to define high-angle ‘grain’ boundaries. The value of 10° is indeed commonly applied to define grain boundaries from EBSD data acquired on natural minerals^23^, including quartz^67^. However, this ad hoc threshold value is based on the experience of material physicists and geologists, and hence, grain boundaries with lower misorientation angles are not excluded to occur in our samples, particularly considering subgrain boundaries that turned into grain boundaries due to progressive subgrain rotation. To be on the safe side, we therefore applied a misorientation angle of 6° to limit the impact of potential “false” subgrain boundaries, and hence, to ensure that decorated intra-grain boundaries are not, in fact, grain boundaries. KAM and GND maps have been also produced using 6° to define grain boundaries, as well as considering neighbours of order 1 and applying a halfquadratic denoising filter with a smoothing parameter of 0.5.

### Focused ion beam and transmission microscopy

Focused ion beam (FIB) cross-sectioning has been performed at the *Institut de Physique du Globe de Paris* (IPGP, France) and *Institut des Sciences de la Terre d’Orléans* (ISTO, France) using a FEG-SEM from ZEISS, respectively an AURIGA in Paris and a CROSSBEAM 550 in Orléans. Thanks to a Gallium ion beam, this technique allows slide-by-slide milling of a 54°-tilted rock sample over several micrometres deep and wide with a nanometre resolution^68^. Each slide can be observed during the milling using secondary and/or backscattered electron/ion images, giving the opportunity to detect the best location for producing and extracting FIB foils. Except for the high-resolution volume of 10 × 10 × 10 nm^3^ voxel size, all FIB volumes have been produced without drifting corrections and based on SEM image acquisition every 100 to 200 nm. The high-resolution volume has been meanwhile acquired using carbon marks on a platinum pad with the ZEISS Atlas 5 software for drifting corrections. The opensource imageJ software has been then used to compile SEM images and reconstruct the FIB volumes (videos uploaded separately). The high-resolution volume has been finally segmented based on the shades of grey and using the VG-studio software. While FIB foils are located in Fig. 1 and supplementary Fig. 1, the milling zone of the high-resolution volume is located in supplementary Fig. 5.

TEM investigations have been then performed on FIB foils of thicknesses ranging between 150 and 250 nm. Foils were produced at the *Institut de Physique du Globe de Paris* (IPGP, France) or *Institut d’électronique*,* microélectronique et nanotechnologie* in Lille (IEMN, France). TEM observations were then acquired at the *Université Paris Cité* (Paris, France) and MACLE-CVL platform (Orléans, France) using a JEOL ACCELARM 200 CF. At both instances, we applied a voltage of 200 kV and a current of ~ 10 µA, focusing the beam for maximum 30 s. These analytical settings were appropriate to avoid damage of the sample during acquisition, i.e., without amorphizing quartz by radiolysis. Indeed, quartz is known to be highly beam sensitive and tends to amorphize under electron irradiation^69,70^, but by proceeding quickly enough, we were able to acquire lattice fringe images without changing the morphology of the amorphous/crystal interface.

## Supplementary Information

Below is the link to the electronic supplementary material.

Supplementary Material 1

## Data Availability

Videos and figures listed/displayed in the Supplementary Information, as well as the EBSD data source files, are available at the following address: https:/doi.org/10.5281/zenodo.15118370
