# Supplementary material for: Quartz porosity in amorphous SiO2 of granitic shear bands
Source: Sci Rep. 2026 Feb 2;16:6996. doi: 10.1038/s41598-026-37576-x (PMC12920790; doi:10.1038/s41598-026-37576-x)
Supplement: Supplementary file 1 — Supplementary Material 1 [file 41598_2026_37576_MOESM1_ESM.pdf]

Scientific Reports

Supplementary information for

Quartz porosity in amorphous SiO_2 of granitic shear bands

Jacques Précigout[1], Cécile Prigent[2], Gina McGill[1], Laurent Arbaret[1], Laura Airaghi[1] and Mathieu Soret[1,3]

[1]Institut des Sciences de la Terre d'Orléans (ISTO), Univ. Orléans, CNRS, BRGM, UMR7327, Orléans, France
[2]Institut de Physique du Globe de Paris (IPGP), Univ. Paris Cité, CNRS, UMR7154, Paris, France
[3]Laboratoire de Géologie, École Normale Supérieure (ENS) de Paris, CNRS, UMR8538, Paris, France

Contents of the file

This file provides a description of the video parameters used to produce scanning electron microscopy (SEM) stack images acquired during focused ion beam (FIB) milling, as well as six supplementary figures to provide:
(1) additional data on pore distribution with respect to substructures in the quartz-rich shear band;
(2) geometrically necessary dislocation (GND) densities of a decorated quartz-rich shear band using both 'conventional' and high-angular resolution (HR-) EBSD;
(3) locations of FIB foils and FIB areas where videos have been produced, including further EBSD maps of Kernal Average Misorientations (KAM);
(4) Scanning transmission electron microscopy (STEM) images of one FIB foil that includes a low-angle inner boundary where Moiré interference patterns have been observed, as well as micropores partly embedded into bright layers of (amorphous) SiO_2;
(5) a STEM image of a FIB foil revealing FIB-induced damages;
(6) location of the grain boundary where the high-resolution FIB volume reconstruction has been produced (also shown in Video #12).

Additional Supplementary Information (available at https://doi.org/10.5281/zenodo.15118370)

- Video #1
- Video #2
- Video #3
- Video #4
- Video #5
- Video #6
- Video #7
- Video #8
- Video #9
- Video #10
- Video #11
- Video #12

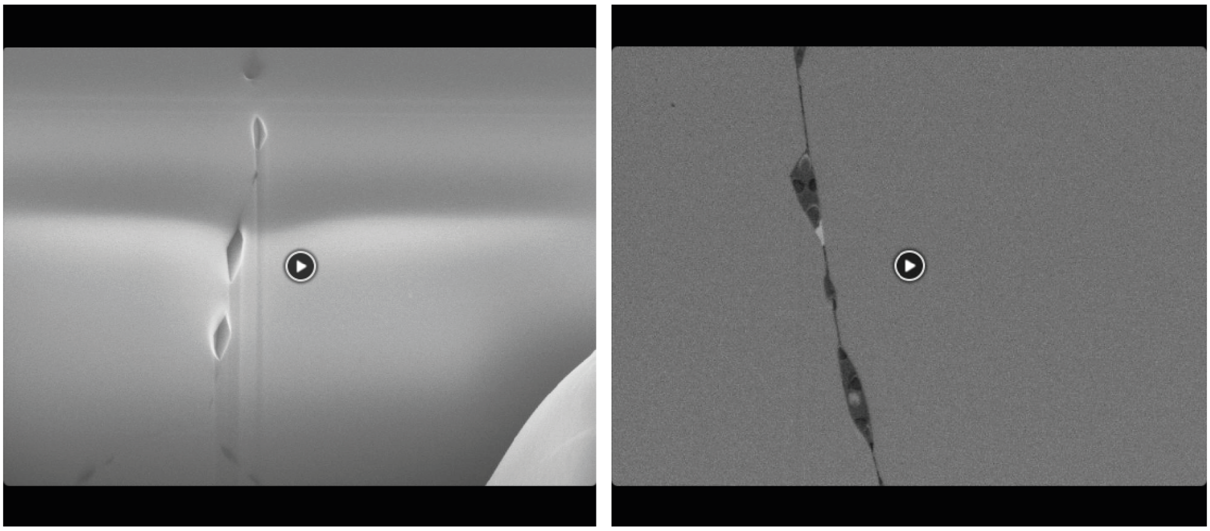

Video files | Stacked SEM images of porosity within FIB volumes acquired along decorated grain boundaries and intra-grain substructures. Twelve videos have been produced and located in supplementary figure 3. Except for the Video #12, all SEM images have been acquired during the FIB milling every 100 or 200 nm and using the SESI detector in a Zeiss AURIGA. The Video #12, on the other hand, has been produced using SEM images acquired every 10 nm with the ESB detector in a Zeiss CROSSBEAM 550. For drifting corrections, the acquisition has been monitored using the Atlas 5 software and carbon marks on a platinum pad at the sample surface (see supplementary figure 6). All images have been then compiled using the imageJ software to produce the videos. Images have a pixel size of 10×10 nm^2, so that the scale can be deduced from the video dimension written in pixels in the file name. The latter also indicates the type of boundary, i.e., grain or intra-grain boundaries, including subgrain and inner boundaries.

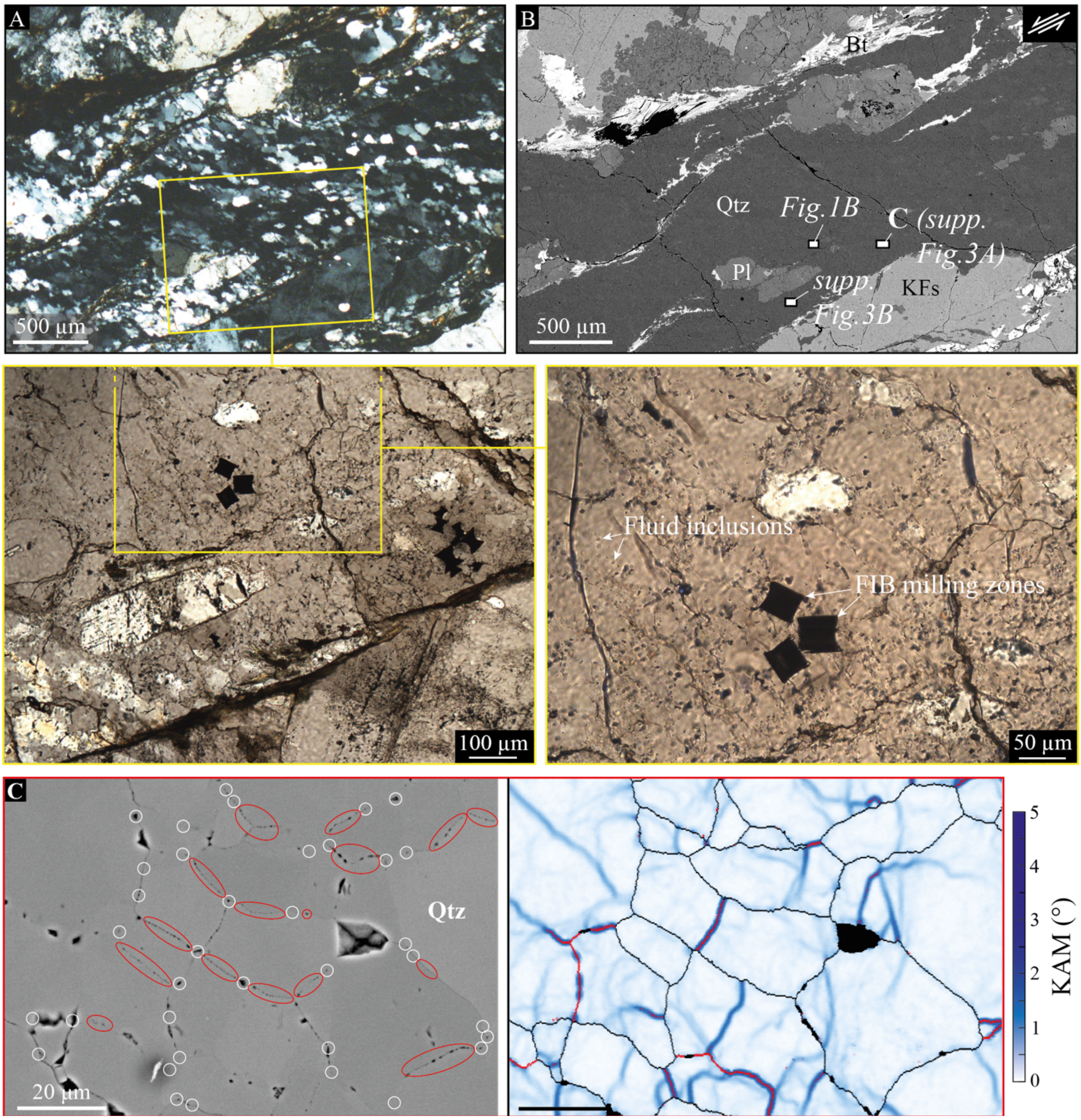

Supplementary Figure 1 | Location of SEM images and additional data of pore distribution with respect to quartz substructures. (A) Cross-polarized optical image of the quartz-rich shear band where the investigations have been performed. Zoom optical images (natural light) are photographs of the main area investigated using FIB. They give rise to the multitude of fluid inclusions found in some areas of quartz-rich shear bands. **(B)** Backscattered electron (BSE) image of the shear band (modified after Précigout *et al.*[11]), providing the locations of the images shown in figure 1 of the manuscript and in supplementary figure 3. The sample is a geological thin section cut perpendicular to the foliation (Z) and parallel to the lineation (X). The sense of shear is sinistral. Qtz = quartz; Pl = plagioclase; KFs = K-feldspar; Bt = biotite. **(C)** Additional BSE image and its companion KAM map highlighting pores at the intersection point between grain boundaries and substructures (white circles), as well as pores that do no intersect with any substructure (red circles/ellipses). While subgrain boundaries are shown in red on the KAM map, grain boundaries and non-indexed pixels are shown in black.

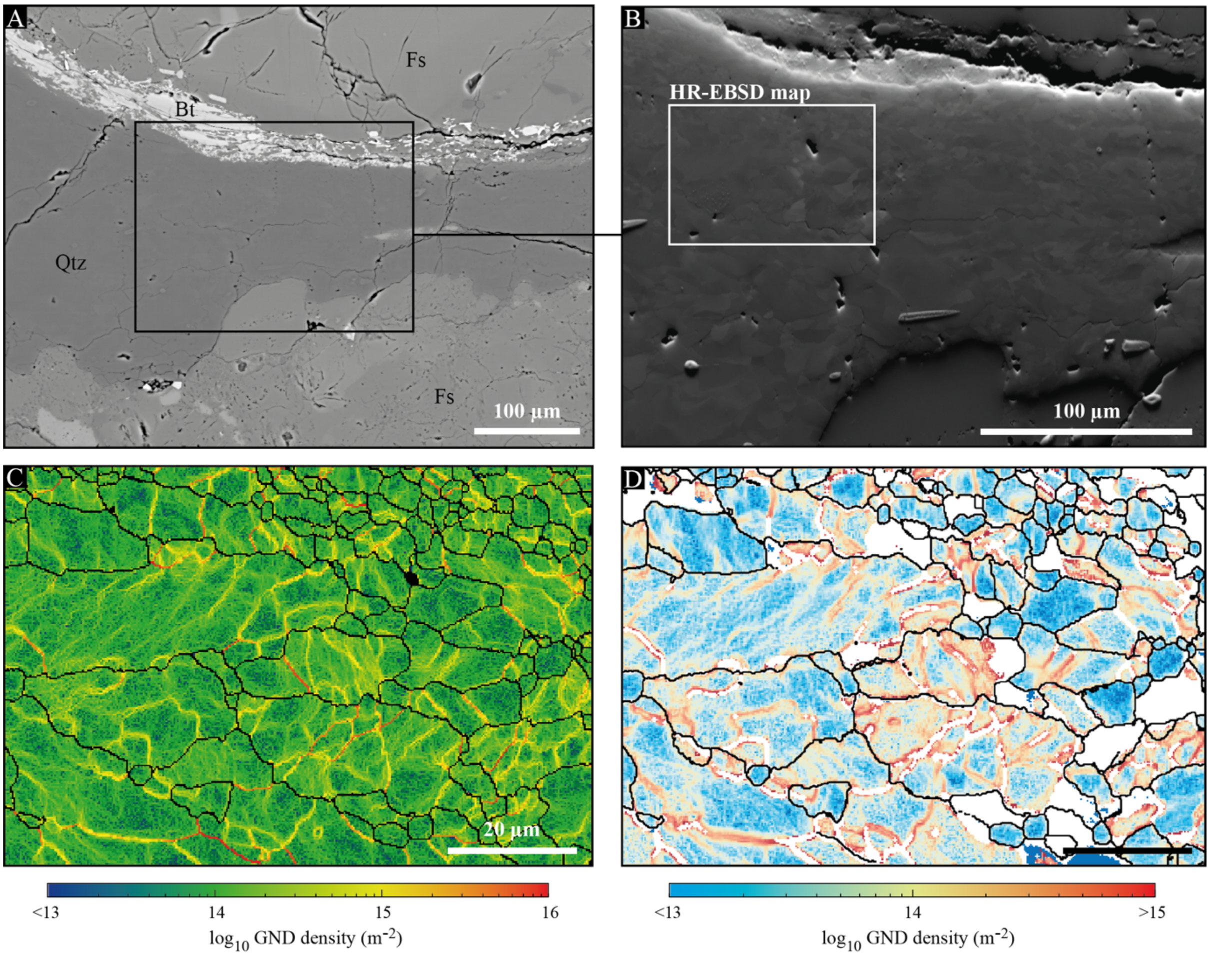

Supplementary Figure 2 | High-angular resolution (HR-)EBSD map of a mylonitic quartz-rich shear band from Naxos. (A) BSE image of the shear band. The image is oriented in the XY structural plane. Qtz = quartz; Bt = Biotite; Fs = Feldspar. **(B)** Forescattered electron image of the close-up area shown in **A**. Using forescatter detectors on the EBSD camera in a scan mode, such an image better reveals quartz grains based on their misorientation with respect to their neighbouring grains. **(C)** GND densities for quartz, as deduced from a conventional EBSD map located in **B**. Grain boundaries and non-indexed points are in black. **(D)** GND densities for quartz of the same area, as deduced from HR-EBSD data. The map has been acquired at the University of Cambridge using a FEG-SEM from FEI (Quanta-650F), and then post-treated using the cross-correlation of diffraction patterns, details of which are given in Wallis *et al.*[24]. White areas indicate where the cross-correlation was not possible. One may notice that HR-EBSD predicts dislocation densities higher than 10^{15} m^{-2} along some inner boundaries. In some cases, the lattice curvature gradients were even so high that the cross-correlation was not possible.

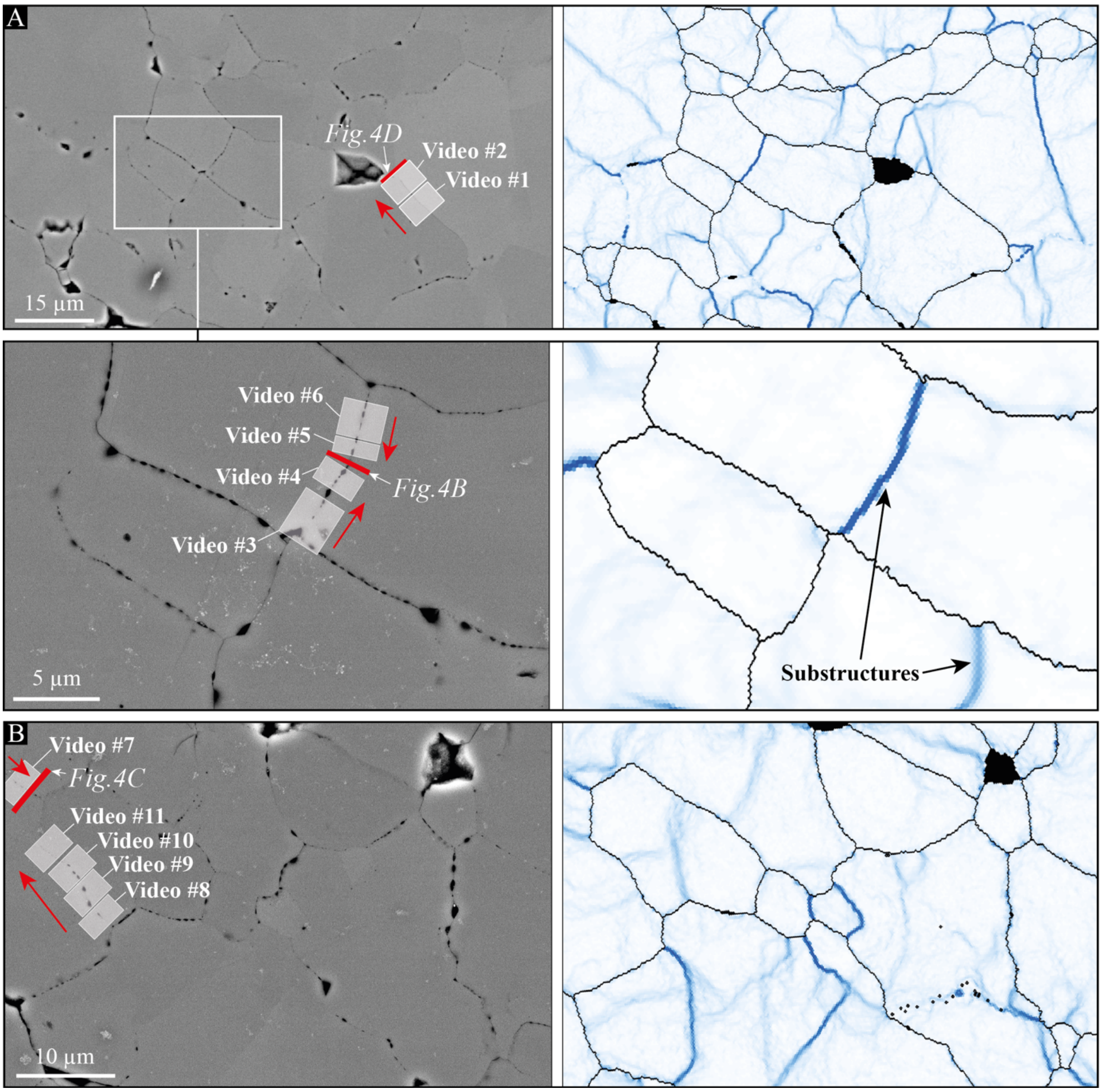

Supplementary figure 3 | Location of FIB foils and reconstructed FIB volumes (videos). **(A, B)** SEM images (backscattered electron) locating FIB foils (red thick lines) and FIB volume areas (transparent white boxes) along grain and intra-grain 'substructures' boundaries. While the red arrows indicate the sense of milling, the figure numbers refer to the figures of the manuscript. Each image is also accompanied by an EBSD map plotting KAM over the same area. The KAM color bar is given in supplementary figure 1. The EBSD maps have been here acquired using an EDAX pegasus system (TSL-OIM software) on a Tescan FEG-SEM from the BRGM (France). The analytical conditions and post-treatment details are given in Précigout *et al.*[51,53].

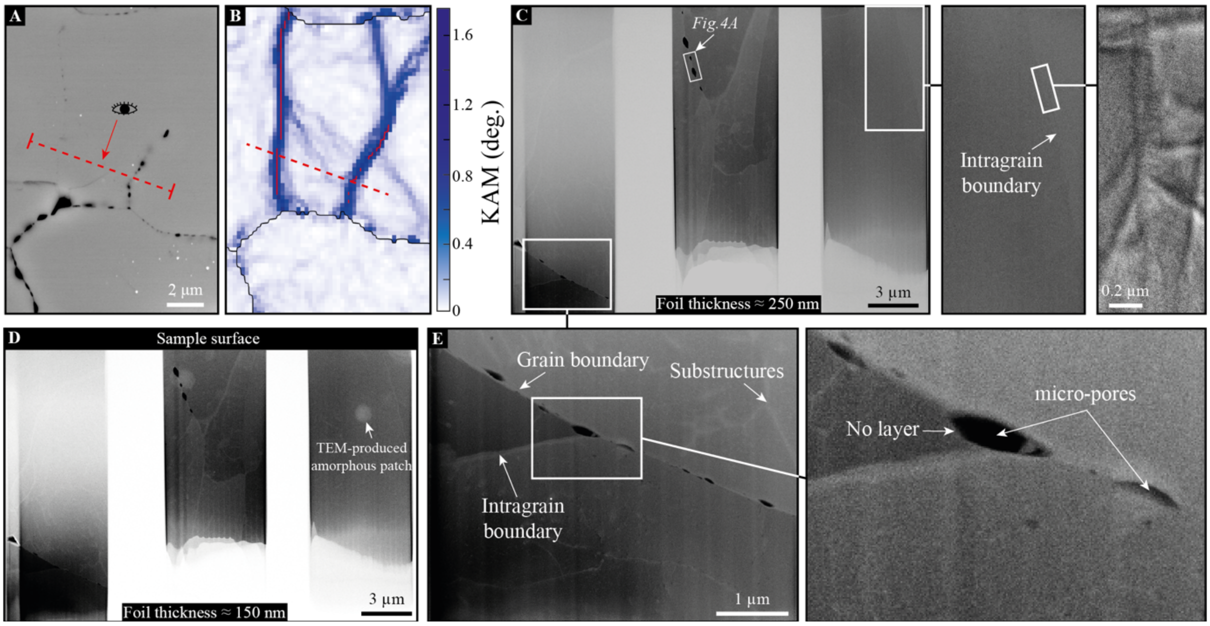

Supplementary Figure 4 | Porosity distribution and quartz substructures, as revealed by STEM images of a FIB foil. (A) Location of the FIB foil (dotted red line) on a SEM image (backscattered electron). The foil is also located in figure 1 of the manuscript. **(B)** KAM map of the area shown in **A**. Grain and subgrain boundaries are respectively shown in black and red. **(C)** STEM (scanning transmission electron microscopy) image of the FIB foil using a FEG-SEM (Strata DB 235 from FEI). The analytical conditions were 4 kV and 200 pA at a working distance of 5.1 mm. The foil, here of around 250 nm thick, highlights grain and intra-grain boundaries decorated (or not) by micropores. While most of the boundaries are wetted by a light grey layer (amorphous), some others (here an inner boundary) are characterized by Moiré interference patterns, suggesting that no amorphous – or very little – material is present (right panels). The figure also gives the location of the figure 4A in the manuscript. **(D)** STEM image of the same FIB foil thinned at around 150 nm thick. The figure shows that substructures did not change significantly during the thinning. One may also notice the presence of an amorphous patch that we intentionally produced by focusing the electron beam at the TEM. **(E)** Close-up of the intersection between a decorated grain boundary and an intra-grain boundary (located in **C**). Both boundaries are wetted by a light grey (possibly amorphous) layer, which partially embeds some micropores that adopt an angular shape where the layer is absent.

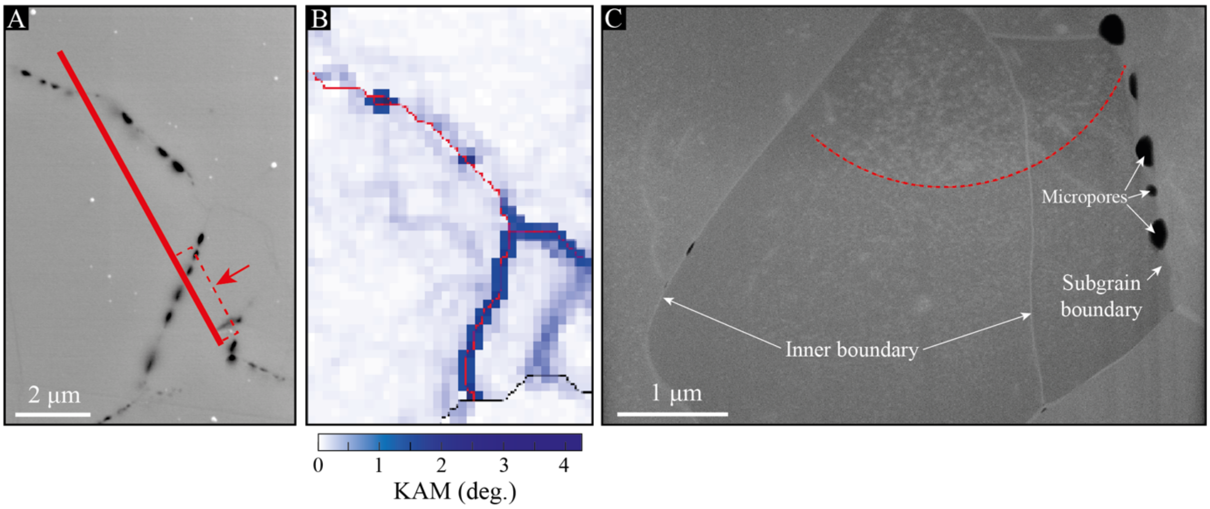

Supplementary Figure 5 | Evidence of an artefact produced during the FIB milling. (A) Location of the FIB foil (red thick line) on a SEM image (backscattered electron). The image is located in figure 1D of the manuscript. **(B)** KAM map over the same area. Grain and subgrain boundaries are respectively shown in black and red. **(C)** STEM image of the FIB foil using a SEM (Strata DB 235 from FEI). The field of view and direction of observation are respectively indicated in **A** by a red dotted line and a red arrow. The STEM image provides a detailed view of micropores and substructures, including inner and subgrain boundaries wetted by light grey (amorphous?) SiO_2. The image also highlights different densities of "white spots" that describe a circular arc (dotted red line), suggesting a FIB artefact related to beam damages of the foil surface. These spots correspond to the irregular loop-shape microstructures on TEM images shown in figure 4 of the manuscript.

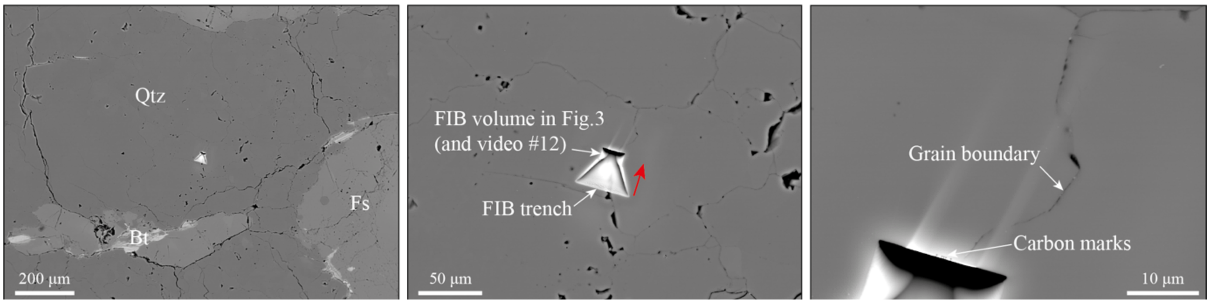

Supplementary Figure 6 | Location of the high-resolution FIB volume. From left to right, SEM images (backscattered electron) that locate the area where the FIB volume has been extracted to produce the high-resolution video #12 of micropores along an open grain boundary. The quartz-rich shear band is part of the same sample close to the shear band shown in supplementary figure 1. During the milling along the grain boundary, SEM images have been acquired every 10 nm, and then stacked to produce the video, as well as the volume reconstruction displayed in figure 3 of the manuscript. The red arrow indicates the sense of milling. On the right image, we can see carbon marks that have been used for drifting corrections. Qtz = quartz; Fs = Feldspar; Bt = Biotite.
